# Thoracic Tumor Volume Delineation in 4D-PET/CT by Low Dose Interpolated CT for Attenuation Correction

**DOI:** 10.1371/journal.pone.0075903

**Published:** 2013-09-26

**Authors:** Tzung-Chi Huang, Yao-Ching Wang, Chia-Hung Kao

**Affiliations:** 1 Department of Biomedical Imaging and Radiological Science, China Medical University, Taichung City, Taiwan; 2 Division of Radiation Oncology, China Medical University Hospital, Taichung City, Taiwan; 3 Department of Nuclear Medicine, China Medical University Hospital, Taichung City, Taiwan; University of Navarra, Spain

## Abstract

**Purpose:**

4D-PET/CT imaging is an excellent solution for reducing the breathing-induced effects in both CT and PET images. In 4D-PET/CT, 4D-CT images are selected to match those of 4D-PET phase by phase and the corresponding phases are used for attenuation correction in 4D-PET. However, the high radiation dose that patients acquire while undergoing 4D-CT imaging for diagnostic purposes remains a concern. This study aims to assess low-dose interpolated CT (ICT) for PET attenuation correction (PET_ICT_) in thoracic tumor volume delineation.

**Methods and Materials:**

Twelve thoracic cancer patients (10 esophageal and 2 lung cancer cases) were recruited. All patients underwent 4D-PET/CT scans. The optical flow method based on image intensity gradient was applied to calculate the motion displacement in three dimensions for each voxel on two original extreme CT phases in the respiratory cycle, end-inspiration and end-expiration. The interpolated CTs were generated from two phases of the original 4D-CT using motion displacement.

**Results:**

Tumor motion due to respiration was estimated in the anterior-posterior dimension, the lateral dimension and the superior-inferior dimension by the optical flow method. The PET_ICT_ and ICT (4D-PET _ICT_/ICT) matched each other spatially in all the phases. The distortion of tumor shape and size resulting from respiratory motion artifacts were not observed in 4D-PET_ICT_. The tumor volume measured by 4D-PET _ICT_/ICT correlated to the tumor volume measured by 4D-PET/CT (*p* = 0.98).

**Conclusions:**

4D-PET_ICT_ consistently represented the interpretation of FDG uptake as effectively as 4D-PET. 4D-PET _ICT_/ICT is a low-dose alternative to 4D-CT and significantly improves the interpretation of PET and CT images, while solving the respiratory motion problem as effectively as 4D-PET/CT.

## Introduction

Positron emission tomography/computed tomography (PET/CT) enables the precise localization and characterization of sites of radiotracer uptake for tumor detection. In imaging, the hybrid scanners combine anatomic and functional information to improve cancer diagnosis, tumor staging and restaging, and tumor response under treatment. They also aid in target volume delineation in radiotherapy treatment planning, which is required to calculate the optimal radiation dose for the tumor and the surrounding normal tissues [1,2]. Therefore, PET/CT has been widely used in routine clinical practice.

Tumor motion due to respiration creates challenges in PET and CT imaging and makes PET/CT imaging more difficult [3,4]. Motion artifacts on CT imaging not only introduce errors in localization and the overestimation of tumor volume due to blurring of the tumor volume, but can also cause the underestimation of standardized uptake value (SUV) in PET quantification [5-8]. In PET/CT imaging of thoracic cancer patients, the mis-registration in fusion images between PET and CT because of respiratory motion is still a major problem. In addition, the difference in acquisition time between PET (15 minutes) and CT (10 seconds), which causes spatial misalignment, is another concern when PET/CT imaging is performed [9]. PET/CT imaging that uses Real-Time Position Management (RPM, Varian Medical Systems, Palo Alto, CA, USA), a respiratory motion monitoring system, is referred to as 4D (gated) PET/CT imaging and is an excellent solution for reducing breathing-induced effects on both CT and PET images [10-13]. 4D-CT with gates of equal amplitude, which divide the respiratory signal with reference to the magnitude of the breathing signal by RPM, is currently the one of the most optimal equipment for capturing lung motion and is frequently used for motion correction in PET. In 4D-PET/CT, 4D-CT images are selected to match those of the 4D-PET phase by phase and are used for attenuation correction in the corresponding 4D-PET. Nehmeh et al reported that 4D PET/CT increases the accuracy of the coregistration of PET and CT, the SUV and the accuracy of tumor volume calculation in PET [14]. Pan et al derived the average CT (ACT) from 4D-CT for attenuation correction to achieve better matching between PET and CT and also, to reduce the SUV loss due to respiration [15]. Although previous studies have presented excellent methods for correcting image artifacts from respiration, the high radiation dose acquired by patients from diagnostic 4D-CT remains a concern.

We previously developed interpolated CT (ICT), which is generated from the end-expiration and end-inspiration phases of CT and interpolated phases using deformable image registration, for PET attenuation correction (AC). This allows for the correction of misalignment artifacts and SUV quantification errors for thoracic tumors with high dose reduction [16]. Its clinical merits in six oncologic patients and its potential application in cardiology have also been reported [17]. ICT has been reported to be a robust, accurate, low-dose alternative to 4D-CT and it works well for a large range of respiratory motion amplitudes [18]. This study aims to assess the interpretation of 4D-PET using ICT for AC on 4D-PET/CT in the spatial matching of PET and CT, localization of tumors, quantification of tumor volume and quantitation of SUV for clinical thoracic cancer patients.

## Materials and Methods

### Patients

Twelve thoracic cancer patients (10 esophageal and 2 lung cancer cases) were retrospectively recruited in this study. All patients were male and ranged in age from 37 to 63 years, with a median age of 53 years. The tumors were mostly in the lower section of the esophagus and lower lobe of the lung, and consisted of squamous cell carcinoma and dysplastic squamous cells. The patients’ clinical characteristics are listed in Table 1. Written informed consent was obtained from all patients, and the collection of clinical patient data was approved by the ethics committees of the China Medical University Hospital, Taiwan (DMR98-IRB-171-2).

**Table 1:** The clinical characteristics of patients.

Patient #	Histology	Location	Tumor motion (mm)			Type	Stage
			AP	LAT	SI		
1	Squamous cell carcinoma	Esophageal cancer (Lower)	3.01	1.75	3.48	Primary	ii
2	Squamous cell carcinoma	Esophageal cancer	0.95	1.15	2.63	Primary	ii
3	Squamous cell carcinoma	Esophageal cancer (Upper)	1.33	0.93	5.34	Primary	ii
4	Squamous cell carcinoma	Esophageal cancer (Middle)	5.28	6.13	5.36	Lymph node	iii
5	Squamous cell carcinoma	Esophageal cancer (Lower)	2.82	2.25	3.32	Lymph node	iii
6	Dysplastic squamous cells	Esophageal cancer (Middle to lower)	2.51	1.04	1.54	Lymph node	iii
7	Squamous cell carcinoma	Esophageal cancer (Lower)	2.67	5.35	7.81	Lymph node	iv
8	Squamous cell carcinoma	Esophageal cancer (Lower)	1.59	0.50	1.14	Lymph node	iv
9	Squamous cell carcinoma	Esophageal cancer (Upper)	1.06	1.13	1.84	Lymph node Lymph node	ii
10	Squamous cell carcinoma	Esophageal cancer (Lower)	2.89	1.87	4.49		iv
11	Squamous cell carcinoma	Lung cancer, Left lower lobe	3.49	1.78	3.85	Lymph node	iii
12	Squamous cell carcinoma	Lung cancer, Right lower lobe	2.54	1.70	4.18	Lymph node	iii

Abbreviations: AP = anterior-posterior, LAT = lateral, SI = superior-inferior

### 4D-PET/CT

**Figure 1:**
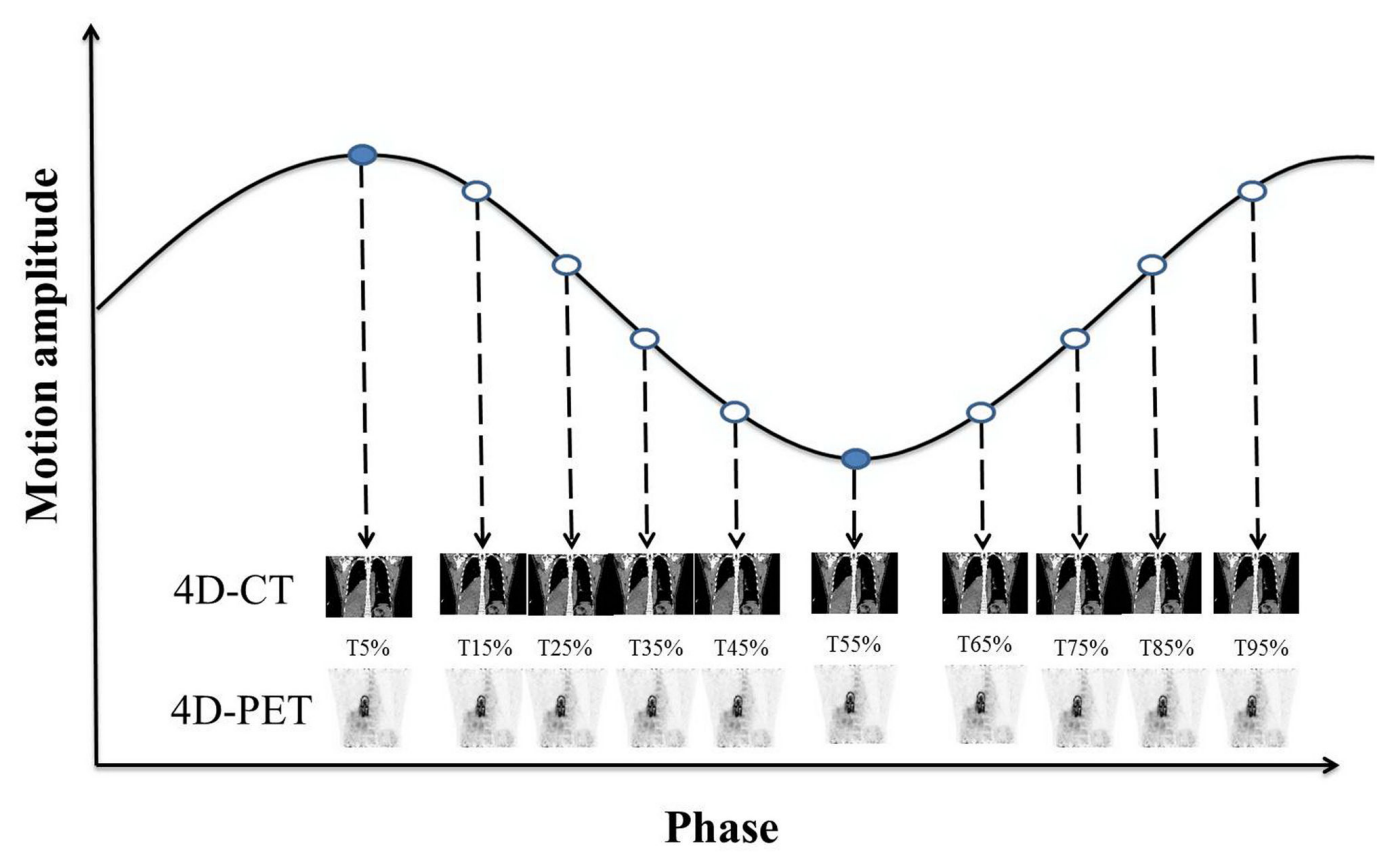
4D-CT and 4D-PET imaging. The respiratory cycle was divided into 10 phases based on motion amplitude. The 10 respiratory phases were labeled T5%, T15%, … T95%, with the T5% phase corresponding approximately to the normal end-inspiration and T55% to the end-expiration for both CT and PET. 4D-CT was used for attenuation correction of 4D-PET on a phase-by-phase basis so that they would match each other spatially.

The 4D-PET/CT data were acquired using GE PET/CT-16 slice, Discovery STE (GE Medical Systems, Milwaukee, Wisconsin, USA), incorporated with Varian real-time position management (RPM system, Varian Medical Systems, Inc. Palo Alto, CA) for respiratory motion tracking. For 4D-CT, sine-mode scanning was used to provide images of 10 phases of a breathing cycle. The CT image in each phase was binned according to the RPM respiratory signal based on the motion amplitude. The 4D sinograms were reconstructed on a 512 x 512 image matrix. The pixel size in the transaxial slice of the 4D CT images was approximately 0.98x0.98 mm^2^, and the slice thickness was 2.5 mm. The 10 respiratory phases were labeled as T5%, T15%, … T95% phases, with the T5% phase corresponding approximately to the normal end-inspiration and the T55% to the end expiration, as illustrated in Figure 1.

FDG with an activity of approximately 10 mCi was administered to the patients, and the PET/CT scan session was performed approximately 40 minutes after injection. 4D-PET was acquired in 10 bins in synchrony with the breathing cycle during gated PET acquisition using RPM to monitor the respiration, as is the case in 4D-CT. PET images were reconstructed on a 128 x 128 image matrix with a voxel size of 4.46 x 5.46 x 3.27 mm^3^. The ordered subset expectation maximization reconstruction method was used with 2 iterations and 20 subsets. For the scans, a post-reconstruction Gaussian filter was applied with a full-width at half-maximum of 7 mm. 4D-CT scan was used for attenuation correction of 4D-PET on a phase-by-phase basis, such that they matched each other spatially, as show in Figure 1.

### Interpolated average CT (ICT) and PET_ICT_

The optical flow method (OFM), a deformable image registration algorithm based on the image intensity gradient, was applied to calculate the motion displacement in three dimensions for each voxel on two original extreme CT phases in the respiratory cycle, end-inspiration (phase T5%) and end-expiration (phase T55%) [16,18]. The interpolated phases were generated using the deformation matrix from the OFM registration. The total motion displacement field for each voxel in the forward motion map was divided equally into five intervals, resulting in four sets of interpolated CT (ICT) images representing the mid-phases from inspiration to expiration. Similarly, the backward motion map was used to calculate the four mid-phases from expiration to inspiration. Then, PET_ICT_ was acquired by its own-phase ICT with attenuation correction. The two original, plus the eight interpolated phases, composed a complete respiratory cycle, as illustrated in Figure 1.

### Tumor volume evaluation

The tumor volume in PET was defined for each of the ten phases of the 4D-PET and 4D-PET_ICT_. The tumor volumes in PET were manually identified by an experienced physician and all the delineations were manually performed in all phases. In addition, each phase of the PET image was superimposed with the corresponding CT phase to investigate the misalignment between the PET and CT images. The difference in tumor volume between 4D-PET and 4D-PET_ICT_ was compared phase by phase, except in the two original phases (T5% and T55%). We also defined the percentage difference (PD) in the tumor volume as follows:

$$PD=\frac{TumorVolume_{4D-PET}-TumorVolume_{4D-PET_{ICT}}}{TumorVolume_{4D-PET}}\times 100$$

## Results

**Figure 2:**
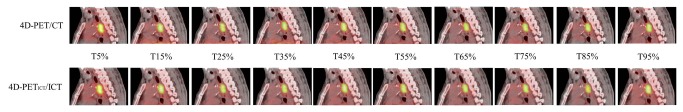
Sagittal views of PET and CT fusion images across the ten phases of a complete respiration cycle. One example for 4D-PET/CT and 4D-PET _ICT_/ICT are shown from patient 3.

**Figure 3:**
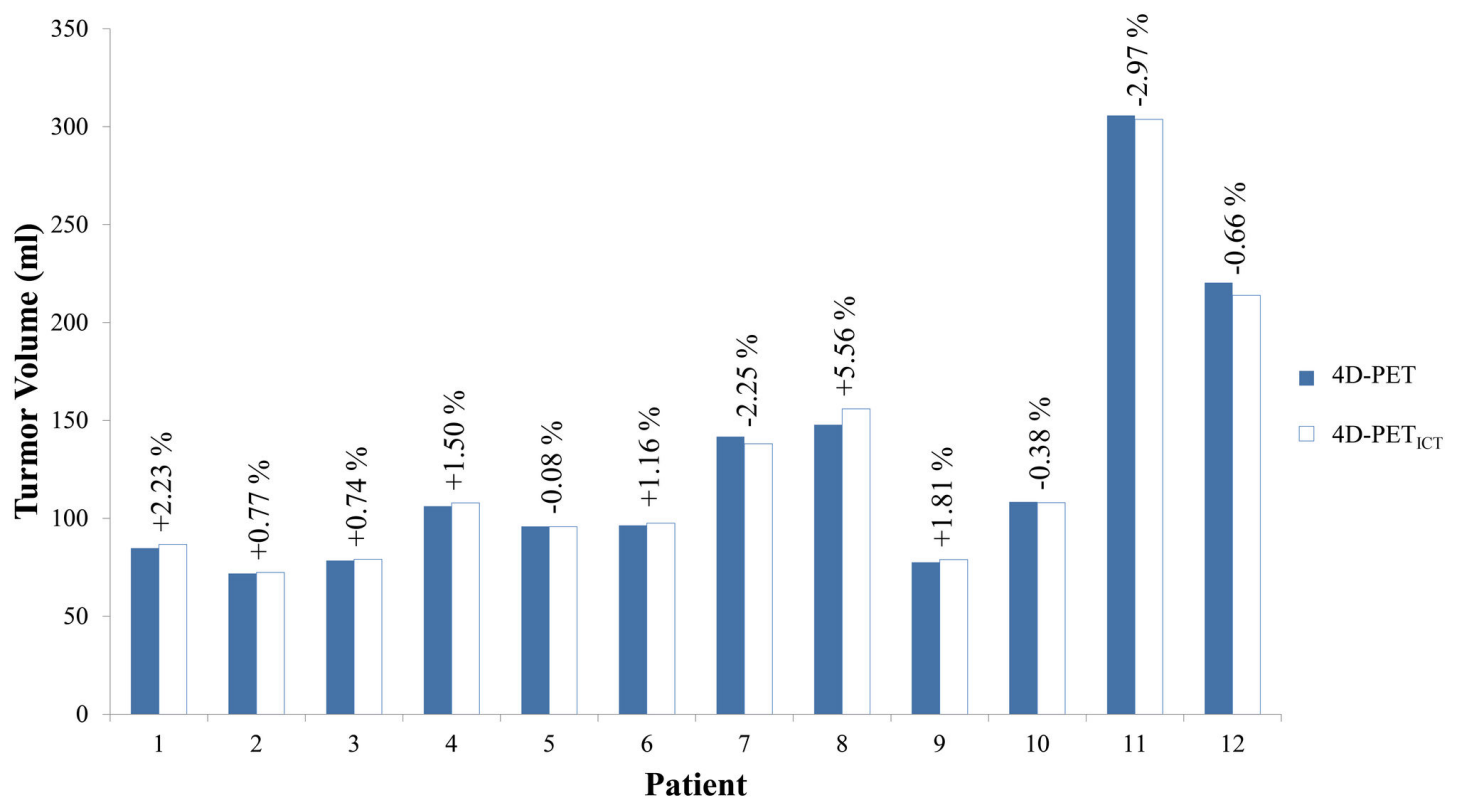
A bar graph showing the mean tumor volume from 4D-PET and 4D-PET_ICT_ for each patient. The percentage difference between 4D-PET and 4D-PET_ICT_ is indicated on the top of each bar.

**Figure 4:**
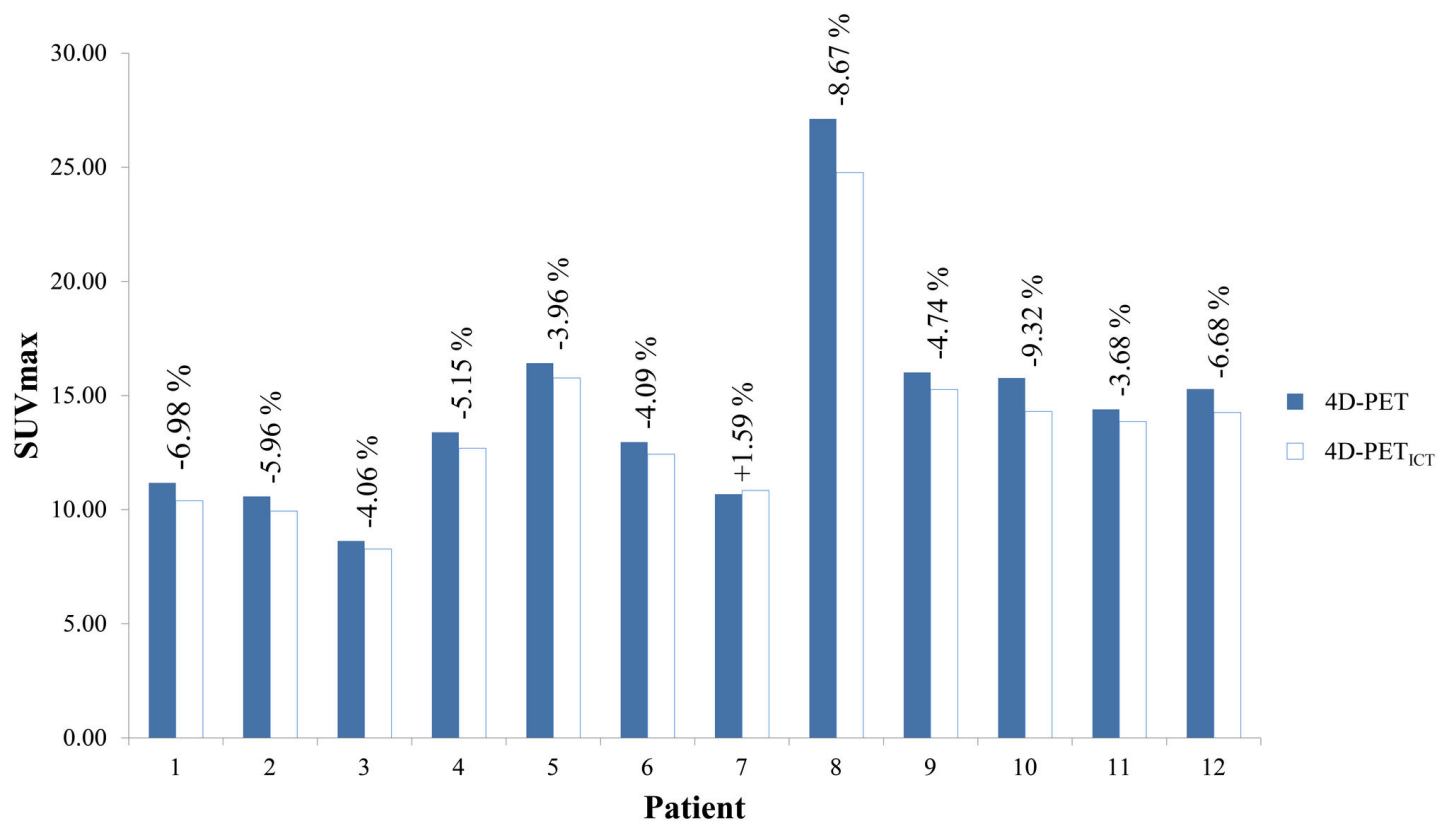
A bar graph representing the SUV_max_ from 4D-PET and 4D-PET_ICT_ for each patient. The percentage difference between 4D-PET and 4D-PET_ICT_ is indicated on the top of each bar.

Tumor motion due to respiration was estimated in the anterior-posterior dimension, the lateral dimension and the superior-inferior dimension by the optical flow method (Table 1). Figure 2 illustrates the sagittal views of PET and CT fusion images across the ten phases of a complete respiration cycle. One example of 4D-PET/CT and 4D-PET _ICT_/ICT from patient 3 is shown. The PET_ICT_ and ICT (4D-PET _ICT_/ICT) in all the phases matched each other spatially. The distortion of tumor shape and size resulting from respiratory motion artifacts was not observed. 4D-PET_ICT_ consistently represented the interpretation of FDG uptake to a similar extent as 4D-PET. Table 2 shows the percentage difference (PD) in tumor volume observed between the 4D-PET and the 4D-PET_ICT_ by phase. The average PD in tumor volume ranged from 0.76% (patient 2) to 5.79% (patient 9). Figure 3 shows a bar graph of the average tumor volumes from 4D-PET and 4D-PET_ICT_ for each patient, with the percentage difference between two volumes shown on top of the bars. Positive PD values represented a larger 4D-PET_ICT_ volume in most cases. SUV_max_ is the threshold level for tumor detection, according to its fluorodeoxyglucose (FDG) uptake. Figure 4 shows the average SUV_max_ from 4D-PET and 4D-PET_ICT_, with the percentage difference shown on the top of bar. The SUV_max_ was smaller on 4D-PET_ICT_ than 4D-PET, with the exception of patient 9.

**Table 2:** The tumor volume from 4D-PET and 4D-PET_ICT_ for each phase, and the results of t tests comparing 4D-PET and 4D-PET_ICT_.

**Patient #**	**Tumor Volume (mL)**	**T15%**	**T25%**	**T35%**	**T45%**	**T65%**	**T75%**	**T85%**	**T95%**	**Average**
1	4D-PET	85.15	86.99	84.67	83.35	83.35	85.05	85.23	84.35	84.77
	4D-PET_ICT_	88.57	87.68	86.02	85.07	87.02	85.99	86.95	85.97	86.66
	PD	4.02	0.79	1.59	2.06	4.40	1.11	2.02	1.92	2.24
2	4D-PET	71.27	72.07	71.89	71.91	72.02	71.41	71.82	72.1	71.81
	4D-PET_ICT_	71.88	72.21	72.56	72.45	72.88	71.92	72.26	72.69	72.36
	PD	0.86	0.19	0.93	0.75	1.19	0.71	0.61	0.82	0.76
3	4D-PET	79.03	78.05	76.76	77.46	77.85	79.37	81.61	77.87	78.50
	4D-PET_ICT_	78.63	79.27	79.36	77.47	79.58	79.21	79.43	79.66	79.08
	PD	0.51	1.56	3.39	0.01	2.22	0.20	2.67	2.30	1.61
4	4D-PET	101.79	99.97	111.15	105.55	107.91	105.58	104.09	114.13	106.27
	4D-PET_ICT_	104.89	101.97	111.5	106.24	105.98	106.23	108.09	117.95	107.86
	PD	3.05	2.00	0.31	0.65	1.79	0.62	3.84	3.35	1.95
5	4D-PET	100.64	101.01	100.93	90.39	91.6	92.9	91.7	97.85	95.88
	4D-PET_ICT_	93.12	102.84	102.23	92.51	91.5	91.55	92.93	99.74	95.80
	PD	7.47	1.81	1.29	2.35	0.11	1.45	1.34	1.93	2.22
6	4D-PET	93.24	100.26	96.86	96.17	101.11	93.81	94.23	95.44	96.39
	4D-PET_ICT_	94.14	102.21	97.61	96.67	105.19	94.56	93.64	96.07	97.51
	PD	0.97	1.94	0.77	0.52	4.04	0.80	0.63	0.66	1.29
7	4D-PET	133.63	143.9	140.95	138.88	129.95	147.99	157.49	141.09	141.74
	4D-PET_ICT_	132.91	142.5	139.75	132.57	132.74	146.48	141.33	136.66	138.12
	PD	0.54	0.97	0.85	4.54	2.15	1.02	10.26	3.14	2.93
8	4D-PET	158.32	136.48	142.96	168	144.88	153.72	129.66	147.96	147.75
	4D-PET_ICT_	157.77	144.1	151.42	172.19	155.32	169.27	141.98	155.62	155.96
	PD	0.35	5.58	5.92	2.49	7.21	10.12	9.50	5.18	5.79
9	4D-PET	77.79	78.32	75.58	76.8	77.59	78.87	78.3	77.22	77.56
	4D-PET_ICT_	78.37	79.57	77.13	78.22	79.86	79.74	79.9	78.85	78.96
	PD	0.75	1.60	2.05	1.85	2.93	1.10	2.04	2.11	1.80
10	4D-PET	105.78	118.06	109.99	112.67	104.58	106.05	99.44	110.77	108.42
	4D-PET_ICT_	108.69	112.97	106.47	109.67	101.73	108.87	105.04	110.61	108.01
	PD	2.75	4.31	3.20	2.66	2.73	2.66	5.63	0.14	3.01
11	4D-PET	327.89	302.82	309.82	297.29	298.8	283.59	317.43	308.54	305.77
	4D-PET_ICT_	321.96	295.69	296.34	289.76	310.98	289.88	310.8	314.62	303.75
	PD	1.81	2.35	4.35	2.53	4.08	2.22	2.09	1.97	2.68
12	4D-PET	217.39	217.37	209.33	252.93	217.58	210.56	214.04	223.91	220.39
	4D-PET_ICT_	209.67	201.29	214.38	236.93	214.56	206.81	199.37	227.77	213.85
	PD	3.55	7.40	2.41	6.33	1.39	1.78	6.85	1.72	3.93

## Discussion

Respiratory motion artifacts cause blurring in the detection of FGD update and the resulting tumor size and volume measurement, and also causes the mislocalization of tumors in PET and CT fusion images of PET/CT. Accurate information can be obtained with 4D-PET, as well as by 4D-CT, in 4D-PET/CT when respiration artifacts are present in images of the thorax. In this study, ICT was successfully applied as a low-dose alternative to 4D-CT, and the PET_ICT_ reconstructed by ICT improved the interpretation of PET, while solving the respiratory motion problem as effectively as 4D-PET/CT. No misalignment between PET_ICT_ and ICT was observed. The potential role of 4D-PET _ICT_/ICT in providing accurate anatomic and functional fusion images for delineating tumor volume contours with low radiation doses was investigated by contrasting the tumor volumes calculated by 4D-PET. The tumor volume from 4D-PET _ICT_/ICT was correlated to the tumor volume from 4D-PET/CT (R^2^ = 0.98).

As the reconstruction of PET using CT for temporal correction in PET/CT is necessary, in PET, CT number, representing electron density, is correlated to SUV. In this study, ICT was calculated from the interpolated images to generate PET_ICT_. The loss of CT intensity usually appeared within the interpolated images, especially with large-amplitude respiratory motion. This factor caused a slightly lower SUV_max_ in 4D-PET_ICT_, and there was a 4.88% loss in average percentage difference of SUV_max_ in our study. Furthermore, the strong correlation between SUV_max_ changes and tumor motions was not observed in our patient cohort.

The limitation of this study was the accurate estimation in movement of respiration. In the interpolation of the mid-phase of CT, the equal spacing of total motion displacement was based on the assumption that patient’s breath is smooth and that the amplitude of the breath in a whole respiration cycle follows a sine curve. If the patient does not breathe in such a normal pattern, the present ICT method based on two original phases and the linear interpolation would not be a good representation of real breath. Active breathing control (ABC) devices, which capture the respiratory phases of the patients, might be an alternative approach to ensure that the phase used is appropriate. Another concern, which is regarding the OFM-based motion displacement, is that OFM was derived from the distribution of the apparent velocities of movement in the brightness patterns between the images. As the “smoothness constraint” for the motion component in the direction of the local gradient was applied, when the tumor moves rapidly with respiration, the displacement estimation using OFM is as a consequence, inaccurate [19]. Third, more investigations are required to reconfirm our preliminary findings that were obtained in a limited number of patients.

## Conclusions

In this study, we have evaluated the effectiveness of PET_ICT_ from ICT attenuation correction compared to 4D-PET in thoracic cancer patients. 4D-PET _ICT_/ICT is a low-dose alternative to 4D-CT and significantly improves the interpretation of PET and CT images while solving the respiratory motion problem as effectively as 4D-PET/CT.

## References

[1] Ashamalla H, Rafla S, Parikh K, Mokhtar B, Goswami G et al. (2005) The contribution of integrated PET/CT to the evolving definition of treatment volumes in radiation treatment planning in lung cancer. Int J Radiat Oncol Biol Phys 63(4): 1016-1023. doi:10.1016/j.ijrobp.2005.04.021. PubMed: 15979817. 15979817 10.1016/j.ijrobp.2005.04.021

[2] Messa C, Di Muzio N, Picchio M, Gilardi MC, Bettinardi V et al. (2006) PET/CT and radiotherapy. Q J Nucl Med Mol Imaging 50: 4–14. PubMed: 16557199. 16557199

[3] Wolthaus JW, van Herk M, Muller SH, Belderbos JS, Lebesque JV et al. (2005) Fusion of respiration-correlated PET and CT scans: Correlated lung tumour motion in anatomical and functional scans. Phys Med Biol 50: 1569–1583. doi:10.1088/0031-9155/50/7/017. PubMed: 15798344. 15798344 10.1088/0031-9155/50/7/017

[4] Shimizu S, Shirato H, Kagei K, Nishioka T, Bo X et al. (2000) Impact of respiratory movement on the computed tomographic images of small lung tumors in three-dimensional (3D) radiotherapy. Int J Radiat Oncol Biol Phys 46(5): 1127-1133. doi:10.1016/S0360-3016(99)00352-1. PubMed: 10725622. 10725622 10.1016/s0360-3016(99)00352-1

[5] Ekberg L, Holmberg O, Wittgren L, Bjelkengren G, Landberg T (1998) What margins should be added to the clinical target volume in radiotherapy treatment planning for lung cancer? Radiother Oncol 48(1): 71-77. doi:10.1016/S0167-8140(98)00046-2. PubMed: 9756174. 9756174 10.1016/s0167-8140(98)00046-2

[6] Yaremko B, Riauka T, Robinson D, Murray B, Alexander A et al. (2005) Thresholding in PET images of static and moving targets. Phys Med Biol 50: 5969–5982. doi:10.1088/0031-9155/50/24/014. PubMed: 16333167. 16333167 10.1088/0031-9155/50/24/014

[7] Park SJ, Ionascu D, Killoran J, Mamede M, Gerbaudo VH et al. (2008) Evaluation of the combined effects of target size, respiratory motion and background activity on 3D and 4D PET/CT images. Phys Med Biol 53: 3661–3679. doi:10.1088/0031-9155/53/13/018. PubMed: 18562782. 18562782 10.1088/0031-9155/53/13/018

[8] Huang TC, Wang YC (2013) Deformation effect on SUVmax changes in thoracic tumors using 4-D PET/CT scan. PLOS ONE 8(3): e538886 PubMed: 23516568. 10.1371/journal.pone.0058886 PMC3597593 23516568

[9] Pevsner A, Nehmeh SA, Humm JL, Mageras GS, Erdi YE (2005) Effect of motion on tracer activity determination in CT attenuation corrected PET images: A lung phantom study. Med Phys 32: 2358–2362. doi:10.1118/1.1943809. PubMed: 16121593. 10.1118/1.1943809 28493572

[10] Britton KR, Starkschall G, Tucker SL, Pan T, Nelson C et al. (2007) Assessment of gross tumor volume regression and motion changes during radiotherapy for non-small-cell lung cancer as measured by four-dimensional computed tomography. Int J Radiat Oncol Biol Phys 68(4): 1036-1046. doi:10.1016/j.ijrobp.2007.01.021. PubMed: 17379442. 17379442 10.1016/j.ijrobp.2007.01.021

[11] Aristophanous M, Berbeco RI, Killoran JH, Yap JT, Sher DJ et al. (2012) Clinical utility of 4D FDG-PET/CT scans in radiation treatment planning. Int J Radiat Oncol Biol Phys 82(1): 99-105. doi:10.1016/j.ijrobp.2010.12.060. 10.1016/j.ijrobp.2010.12.060 21377285

[12] Liu HH, Balter P, Tutt T, Choi B, Zhang J et al. (2007) Assessing respiration-induced tumor motion and internal target volume using four-dimensional computed tomography for radiotherapy of lung cancer. Int J Radiat Oncol Biol Phys 68(2): 531-540. doi:10.1016/j.ijrobp.2006.12.066. PubMed: 17398035. 17398035 10.1016/j.ijrobp.2006.12.066

[13] Nehmeh SA, Erdi YE, Ling CC, Rosenzweig KE, Schoder H et al. (2002) Effect of respiratory gating on quantifying PET images of lung cancer. J Nucl Med 43(7): 876-881. PubMed: 12097456. 12097456

[14] Nehmeh SA, Erdi YE, Pan T, Pevsner A, Rosenzweig KE et al. (2004) Four-dimensional (4D) PET/CT imaging of the thorax. Med Phys 31(12): 3179-3185. doi:10.1118/1.1809778. PubMed: 15651600. 15651600 10.1118/1.1809778

[15] Pan T, Mawlawi O, Nehmeh SA, Erdi YE, Luo D et al. (2005) Attenuation correction of PET images with respiration-averaged CT images in PET/CT. J Nucl Med 46: 1481-1487. PubMed: 16157531. 16157531

[16] Huang TC, Mok GS, Wang SJ, Wu TH, Zhang G (2011) Attenuation correction of PET images with interpolated average CT for thoracic tumors. Phys Med Biol 56(8): 2559-2567. doi:10.1088/0031-9155/56/8/014. PubMed: 21444973. 21444973 10.1088/0031-9155/56/8/014

[17] Wu TH, Zhang G, Wang SJ, Chen CH, Yang BH et al. (2010) Low-dose interpolated average CT for attenuation correction in cardiac PET/CT. Nucl Instrum Methods A 619: 361-364. doi:10.1016/j.nima.2009.11.001.

[18] Mok G, Sun T, Huang TC, Vai MI (2011) Interpolated Average CT for Attenuation Correction in PET – A Simulation Study. IEEE Trans Biomed Eng 60(7): 1927-1934. 10.1109/TBME.2013.2245132 23392338

[19] Zhang G, Huang TC, Feygelman V, Stevens C, Forster K (2010) Generation of composite dose and biological effective dose (BED) over multiple treatment modalities and multistage planning using deformable image registration. Med Dosim 35(2): 143-150. doi:10.1016/j.meddos.2009.05.001. PubMed: 19931027. 19931027 10.1016/j.meddos.2009.05.001

